# Prognostic value of right ventricular native T1 mapping in pulmonary arterial hypertension

**DOI:** 10.1371/journal.pone.0260456

**Published:** 2021-11-29

**Authors:** Ryotaro Asano, Takeshi Ogo, Yoshiaki Morita, Akiyuki Kotoku, Tatsuo Aoki, Kyoko Hirakawa, Sayuri Nakayama, Jin Ueda, Akihiro Tsuji, Mark T. Waddingham, Yasutoshi Ohta, Tetsuya Fukuda, Keiko Ohta-Ogo, Hatsue Ishibashi-Ueda, Teruo Noguchi, Satoshi Yasuda

**Affiliations:** 1 Division of Pulmonary Circulation, Department of Cardiovascular Medicine, National Cerebral and Cardiovascular Centre, Osaka, Japan; 2 Department of Advanced Medical Research for Pulmonary Hypertension, National Cerebral and Cardiovascular Centre, Osaka, Japan; 3 Department of Advanced Cardiovascular Medicine, Graduate School of Medical Sciences, Kumamoto University, Kumamoto, Japan; 4 Department of Radiology, National Cerebral and Cardiovascular Centre, Osaka, Japan; 5 Department of Pathology, National Cerebral and Cardiovascular Centre, Osaka, Japan; 6 Department of Cardiovascular Medicine, National Cerebral and Cardiovascular Centre, Osaka, Japan; Scuola Superiore Sant’Anna, ITALY

## Abstract

**Background:**

Right ventricular function is an important prognostic marker for pulmonary arterial hypertension. Native T1 mapping using cardiovascular magnetic resonance imaging can characterize the myocardium, but accumulating evidence indicates that T1 values of the septum or ventricular insertion points do not have predictive potential in pulmonary arterial hypertension. We aimed to elucidate whether native T1 values of the right ventricular free wall (RVT1) can predict poor outcomes in patients with pulmonary arterial hypertension.

**Methods:**

This retrospective study included 30 patients with pulmonary arterial hypertension (median age, 45 years; mean pulmonary artery pressure, 41±13 mmHg) and 16 healthy controls (median age, 43 years) who underwent native T1 mapping. RVT1 was obtained from the inferior right ventricular free wall during end systole.

**Results:**

Patients with pulmonary arterial hypertension had significantly higher native RVT1 than did controls (1384±74 vs. 1217±57 ms, p<0.001). Compared with T1 values of the septum or ventricular insertion points, RVT1 correlated better with the effective right ventricular elastance index (R = −0.53, p = 0.003), ventricular-arterial uncoupling (R = 0.46, p = 0.013), and serum brain natriuretic peptide levels (R = 0.65, p<0.001). Moreover, the baseline RVT1 was an accurate predictor of the reduced right ventricular ejection fraction at the 12-month follow-up (delta -3%). RVT1 was independently associated with composite events of death or hospitalization from any cause (hazard ratio = 1.02, p = 0.002).

**Conclusions:**

RVT1 was predictive of right ventricular performance and outcomes in patients with pulmonary arterial hypertension. Thus, native T1 mapping in the right ventricular free wall may be an effective prognostic method for pulmonary arterial hypertension.

## Introduction

Pulmonary arterial hypertension (PAH), a progressive life-threatening disease of the pulmonary vasculature, can result in increased pulmonary vascular resistance (PVR), elevated pulmonary arterial pressure (PAP), and right ventricular (RV) failure [1]. Despite the development of effective pulmonary vasodilator drugs, the prognosis of patients with PAH remains poor, with progressive RV failure being the primary cause of death [2]. Current guidelines recommend risk assessment using indirect RV failure parameters [3], and RV ejection fraction (RVEF) and RV end-diastolic volume (RVEDV) obtained by cardiac magnetic resonance (CMR) are predictors of poor survival in patients with PAH [4, 5].

Alterations of RV myocardial components (fibrosis, infiltrations, and myocardial edema) are observed in patients with PAH [6–8], and RV remodeling is not purely a result of afterload burden but a combination of genetic factors, inflammation, metabolic alterations, and ischemia [9]. Hence, characteristics of the RV myocardium could be associated with RV performance and progressive RV dysfunction and might be additional prognostic determinants in PAH [7, 10]. However, characterizing the RV myocardium is challenging. For instance, an endomyocardial biopsy is an invasive routine examination, whereas late gadolinium enhancement (LGE) detects regional RV changes but is unsuitable for the detection of diffuse and interstitial myocardial fibrosis, which is generally observed in the relatively thin RV free wall [11, 12]. Therefore, appropriate approaches should be developed to characterize the RV myocardium.

Native T1 mapping, an emerging CMR technique, can quantify the native T1 relaxation time per pixel and is validated to detect tissue changes, such as lipid accumulation, myocardial edema, and fibrosis, in the left ventricle (LV) [13]. A few studies have assessed T1 values in patients with PAH [14, 15]; for example, Saunders et al. have found that native T1 values of the septum, insertion points, and the LV free wall were elevated in patients with PAH, and T1 values of the RV insertion points correlated significantly with hemodynamic parameters but did not contribute to overall mortality prediction [16]. Notably, the RV insertion points and septum are affected by regional mechanical stress through septal wall bouncing. Therefore, in this study, we aimed to investigate the application of T1 values of the RV free wall in the assessment of PAH severity, RV performance, and PAH prognosis.

## Materials and methods

Data collection and volumetric analysis are described in S1 Appendix.

### Study design and participants

We retrospectively investigated 30 patients with PAH who underwent CMR, including quantitative imaging and native T1 mapping, between September 2015 and March 2017. They were diagnosed with idiopathic PAH (n = 18), PAH associated with connective tissue disease (n = 5), portopulmonary hypertension (n = 3), PAH associated with congenital heart disease (n = 3), or drug-induced PAH (n = 1) according to the 2015 European Society of Cardiology/European Respiratory Society guidelines [3]. A total of 16 healthy controls with normal electrocardiography findings and no history of cardiac disease underwent native T1 mapping, and among them, 10 were also evaluated by volumetric analysis. Patient follow-up began on the day of CMR imaging, and they were tracked through outpatient visits every 1–3 months. The primary endpoint was a composite event defined as death or hospitalization from any cause, except for periodic right heart catheterization (RHC) checkups. The secondary endpoints included each event of death, death from right heart failure, all hospitalizations, hospitalization due to right heart failure, and a composite event defined as death or hospitalization due to right heart failure. This study complied with the principles of the Declaration of Helsinki and was approved by the medical ethics review committee of the National Cerebral and Cardiovascular Center (approval no., M30-006-2). The committee waived the requirement to obtain informed consent because the study was a retrospective observational investigation. Use of the “opt-out” approach to consent was approved. A written explanation for using data was provided on the websites. Patients did not provide written informed consent but were allowed to decline participation.

### Native T1 mapping

**Fig 1:**
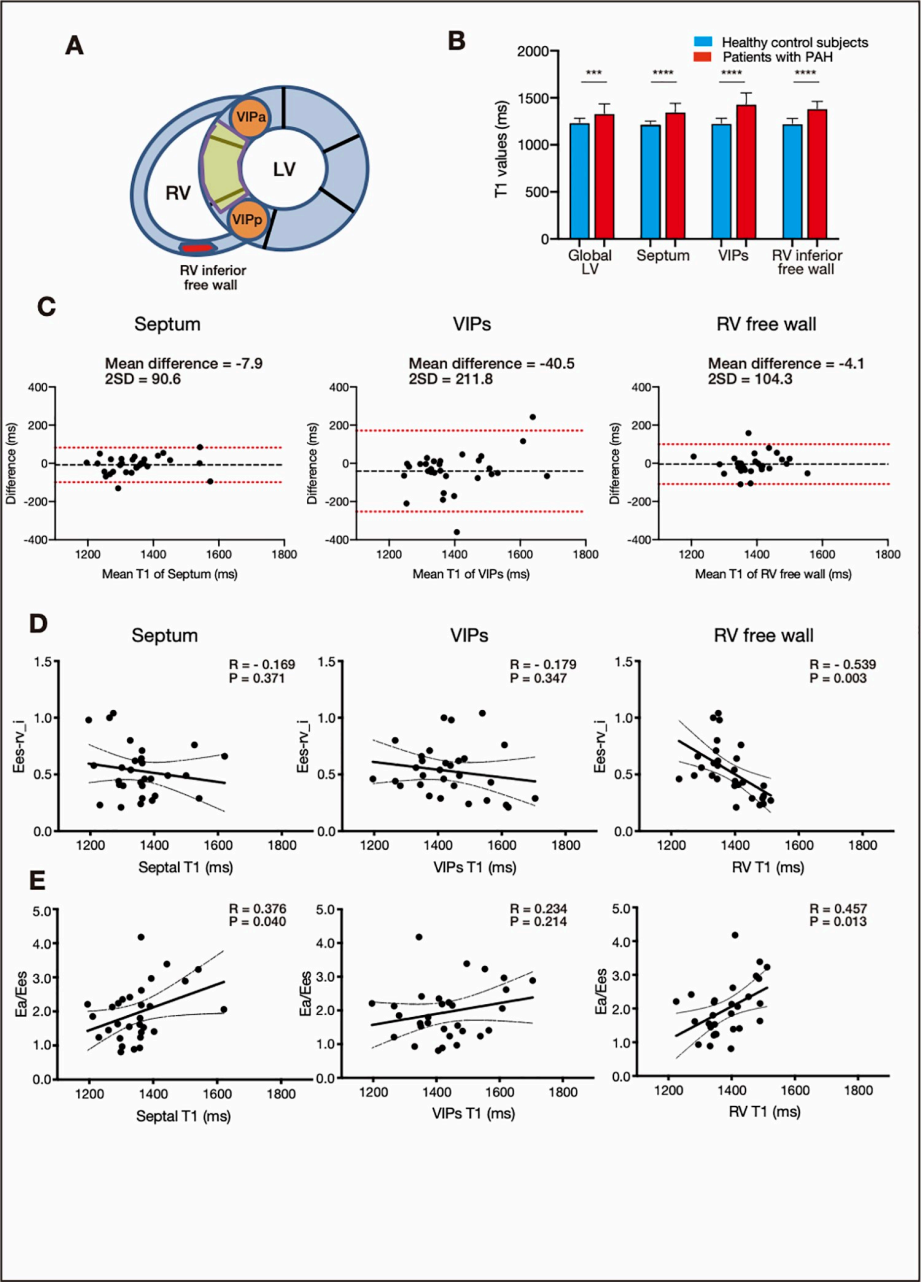
Measurement regions in the right ventricle (RV) and comparisons of T1 values. (a) ROIs of the septum and VIPs are marked in yellow and orange, respectively. ROIs of the RV free wall evaluated in the inferior part are marked in red. (b) Bar plots of native T1 values at each measurement site. The blue and red bars indicate T1 values of patients with PAH and healthy controls, respectively. (c) Interobserver variability analyzed by the Bland–Altman plot. (d) Correlations between T1 values at each measurement site and effective RV elastance using the single-beat method. (e) Correlations between T1 values at each measurement site and RV-pulmonary arterial coupling. Abbreviations: Ea-pa, effective pulmonary arterial elastance; Ees-rv, effective right ventricular elastance; LV, left ventricle; PAH, pulmonary arterial hypertension; ROI, region of interest; RV, right ventricle; VIPs, interventricular insertion points.

CMR was performed using a standardized clinical protocol with a 3.0-T system (MAGNETOM Verio and Vida; Siemens Healthcare GmbH, Erlangen, Germany) [17]. Native T1 mapping was acquired on the mid-ventricular short axis using a modified Look-Locker inversion-recovery (MOLLI) pulse sequence with a 3(3)5 scheme [13]. To capture the thin RV free wall, native T1 mapping was acquired at the end-systolic period with motion correction based on synthetic images [18]. In the MOLLI 3(3)5 scheme, three and five images were acquired after the first and second inversion recovery pulses, respectively, with three recovery beats to allow for complete T1 recovery between inversion recovery pulses. The T1 map was reconstructed using eight source images with different inversion times. Imaging parameters were as follows: echo time, 1.1 ms; repetition time, 2.5 ms; flip angle, 35°; slice thickness, 8 mm; and in-plane resolution, 3.1×1.7 mm^2^. Imaging was performed during suspended respiration. Native T1 values were assessed using regions of interest (ROIs) at the septum, ventricular insertion point (VIP), and RV inferior free wall of the mid-ventricular short-axis T1 maps in the analysis software (Ziostation 2, Ziosoft Inc., Tokyo, Japan). ROIs were traced manually (Fig 1A and S1 Fig) by experienced cardiovascular radiologists and cardiologists to avoid partial volume effects of the surrounding tissues or the blood pool. Furthermore, ROIs were accurately positioned in the inferior part of the RV free wall because of the complex RV anatomy. Native T1 values were calculated as the average of T1 from all pixels for each ROI. Native T1 values of VIPs (i.e., inferior and superior insertion points) were averaged. Interobserver reproducibility was tested for all patients by two independent operators.

### Right ventricular pressure-volume relationships

The single-beat method was employed to assess RV pressure-volume relationships using separately acquired pressure data from RHC and volume data from CMR, as described previously [19, 20]. The median time between RHC and CMR was 4 (interquartile range [IQR], 1–7) days. Briefly, RV maximum pressure was calculated from the nonlinear extrapolation of the early systolic and diastolic portions of the RV pressure curve. End-systolic pressure was approximated as the mean PAP. Effective pulmonary arterial elastance (Ea-pa), a measure of arterial load, was approximated as mean PAP/stroke volume, and RV pressure at end-diastole was assumed to be equal to zero. Effective RV elastance (Ees-RV), a well-validated measure of contractility, was approximated as the slope of the end-systolic pressure-volume relationship. The Ea-pa/Ees-RV ratio describes the ventricular-arterial coupling, and Ea-pa and Ees-RV were indexed for body surface area.

### Serial volumetric cardiac magnetic resonance

Follow-up CMR studies were performed in 18 patients with PAH (13, 2, 2, and 1 with idiopathic PAH, connective tissue disease-associated PAH, portopulmonary hypertension, and congenital heart disease-associated PAH, respectively). We analyzed follow-up CMR imaging over 12 months after baseline CMR imaging (median duration between studies, 570 [IQR, 425–667] days; S2 Fig). According to Bradlow et al., a +3% or −3% change was used to define an increased or decreased RVEF at follow-up, respectively [21].

### Statistical analyses

Data are presented as mean ± standard deviation, median (IQR), or percentage. Student’s t-test was used for between-group comparisons of continuous variables with a normal distribution, whereas the Mann–Whitney U test was used for between-group comparisons of skewed continuous or discrete variables. Nominal variables were compared using the chi-squared test. The strength of correlations was determined using Pearson’s correlation coefficient. The receiver operating characteristic (ROC) curve analysis was used to evaluate the predictive accuracy and cut-off value using the Youden’s index. Univariate Cox proportional hazards analysis was employed to evaluate the relationship between composite outcomes and various selected factors. Variables with a p-value<0.25 in the univariate analysis were subsequently evaluated using a multivariate model with the forward selection method. Kaplan–Meier survival curves were constructed, and the log-rank test was used to compare event-free survival in patients stratified by the risk models. A two-tailed p-value<0.05 was considered statistically significant. Interobserver variability was assessed for native T1 values in all patients using the intraclass correlation coefficient (ICC). All analyses were performed using the Statistical Package for the Social Sciences software version 24.0 (SPSS v24.0; International Business Machines Corp., Armonk, NY, USA) and GraphPad Prism 8 version 8.2.1 (GraphPad, San Diego, CA USA).

## Results

### Demographics and baseline clinical characteristics

As shown in Table 1, this study included 30 patients with PAH (male, 30%; median age at CMR, 45 [IQR, 39–59] years; median disease duration, 74 [IQR, 9–128] months; median brain natriuretic peptide [BNP], 47.5 [IQR, 18.3–81.2] pg/mL).

**Table 1:** Baseline clinical characteristics.

	Variables	PAH	Control	p-values
		(n = 30)	(n = 16)	
	Age (years)	45 (39–59)	43 (30–68)	0.628
	Male sex, n (%)	9 (30)	8 (50)	0.181
	Body mass index (kg/m^2^)	21±6	21±4	0.555
	Idiopathic PAH, n (%)	18 (60)	-	-
	Connective tissue disease associated with PAH, n (%)	5 (17)	-	-
	Portopulmonary hypertension, n (%)	3 (10)	-	-
	Congenital heart disease associated PAH, n (%)	3 (10)	-	-
	Drug-induced PAH, n (%)	1 (3)	-	-
	WHO-FC II/III/IV, n	15/14/1	-	-
	Disease duration (months)	74 (9–128)	-	-
	Six-minute walk distance (m)	480 (387–547)	-	-
	BNP level (pg/mL)	47.5 (18.3–81.2)	-	-
**Medication**				
	PAH-specific therapy	29 (97)	-	-
	Endothelin receptor antagonist, n (%)	26 (87)	-	-
	Phosphodiesterase type-5 inhibitor, n (%)	27 (90)	-	-
	Soluble guanylate cyclase stimulator, n (%)	1 (3)	-	-
	Intravenous epoprostenol, n (%)	15 (50)	-	-
	Oral prostacyclin agonist, n (%)	7 (23)	-	-
**ECG parameters**				
	Heart rate (beat/min)	76±14	-	-
	PR duration (ms)	175±26	-	-
	QRS duration (ms)	114±23	-	-
	QRS axis (degree)	95 (53–107)	-	-
	R/S ratio >1 in V1 (n, %)	18 (60)	-	-
	Right bundle branch block (n, %)	8 (27)	-	-
**Hemodynamics**				
	Right atrial pressure (mmHg)	4 (2–8)	-	-
	Pulmonary capillary wedge pressure (mmHg)	7 (5–8)	-	-
	Systolic PAP (mmHg)	66.8±21.2	-	-
	Diastolic PAP (mmHg)	28.4±10.7	-	-
	Mean PAP (mmHg)	40.8±13.1	-	-
	Cardiac index (L/min/m^2^)	2.9±0.9	-	-
	Pulmonary vascular resistance (Wood units)	9.0±5.4	-	-
**CMR volumetric findings**		(n = 30)	(n = 10)	
	LVEDVi (mL/m^2^)	75.7±21.2	70.2±6.1	0.424
	LVESVi (mL/m^2^)	33.5±12.9	28.1±3.0	0.199
	LV mass index	48.3±8.7	47.4±5.6	0.741
	LVEF (%)	58.7±12.1	59.6±3.0	0.110
	RVEDVi (mL/m^2^)	139.8±69.5	79.0±11.1	0.01
	RVESVi (mL/m^2^)	93.8±58.0	37.6±7.8	0.004
	RV mass index	35.2±12.4	16.8±2.3	0.001
	RVEF (%)	35.9±10.0	52.9±4.6	<0.001

Abbreviations: BNP, brain natriuretic peptide; ECG; electrocardiogram; IQR, interquartile range; LVEDVi, left ventricular end-diastolic volume index; LVEF, left ventricular ejection fraction; LVESVi, left ventricular end-systolic volume index; PAH, pulmonary arterial hypertension; PAP, pulmonary arterial pressure; RVEDVi, right ventricular end-diastolic volume index; RVEF, right ventricular ejection fraction; RVESVi, right ventricular end-systolic volume index; SD, standard deviation; WHO-FC, World Health Organization functional class.

Continuous values are expressed as mean±SD or median (IQR). Categorical values are expressed as number and percentage; n indicates the number of patients.

World Health Organization functional classes (WHO-FC) II, III, and IV were observed in 50%, 47%, and 3% of the patients, respectively. A PAH-specific drug and continuous intravenous epoprostenol were used in 97% and 50% of the patients, respectively. Analysis of hemodynamic parameters demonstrated a mean PAP of 40.80±13.1 mmHg, cardiac index of 2.9±0.9 L/min/m^2^, and PVR of 9.0±5.4 Wood units. CMR imaging data analysis indicated an increased RV end-diastolic volume index (RVEDVi) and RV end-systolic volume index (RVESVi) and a reduced RVEF in patients with PAH compared to healthy controls (RVEDVi: 139.8±69.5 vs. 79.0±11.1 mL/m^2^, p = 0.01; RVESVi: 93.8±58.0 vs. 37.6±7.8 L/m^2^, p = 0.001; RVEF: 35.9±10.0 vs. 52.9±4.6%, p<0.001).

### Clinical parameters and T1 values

As shown in Fig 1B and S1 Table, all native T1 values of the global LV, septum, VIP, and RV free wall (RVT1) were significantly higher in patients with PAH than in healthy controls (global LV: 1334±102 ms vs. 1237±45 ms, p = 0.001; septum: 1350±91 vs. 1219±34 ms, p<0.001; VIP: 1434±118 vs. 1229±53 ms, p<0.001; RV free wall: 1385±75 ms vs. 1226±54 ms, p<0.001; S1 Table). Interobserver ICCs for T1 values of the septum, VIPs, and RV free wall were 0.89, 0.74, and 0.75, respectively, in all patients with PAH (p<0.001, Fig 1C).

Correlations between T1 values at each measurement site and clinical parameters are shown in S2 Table. Septal T1 values correlated with mean PAP, but VIP and RV T1 values did not. Septal and VIP T1 values weakly correlated with the RVEDVi, RVESVi, but not with the RVEF. However, RVT1 had a stronger relationship with the RVEF, as well as the RVEDVi, RVESVi and RV mass index, than did septal and VIP T1 values (RVEF: R = −0.420, p = 0.023; RVEDVi: R = 0.655, p<0.001; RVESVi: R = 0.637, p<0.001; RV mass index: R = 0.622, p<0.001).

The estimated pressure-volume relationship analysis is shown in Fig 1D and S2 Table. Septal and VIP T1 values did not correlate with physiological parameters, but RVT1 was significantly related to Ea-pa/Ees-rv (R = 0.457, p = 0.013) and effective RV elastance index (Ees-rv_i) (R = −0.539, p = 0.003). These findings suggest that RVT1 are more reliable markers for RV performance and adaptation for the afterload burden than are septal and VIP T1 values. Furthermore, RVT1 had strongly positive correlations with serum BNP levels and QRS duration (R = 0.654 and R = 0.720, respectively; p<0.001; S2 Table).

**Fig 2:**
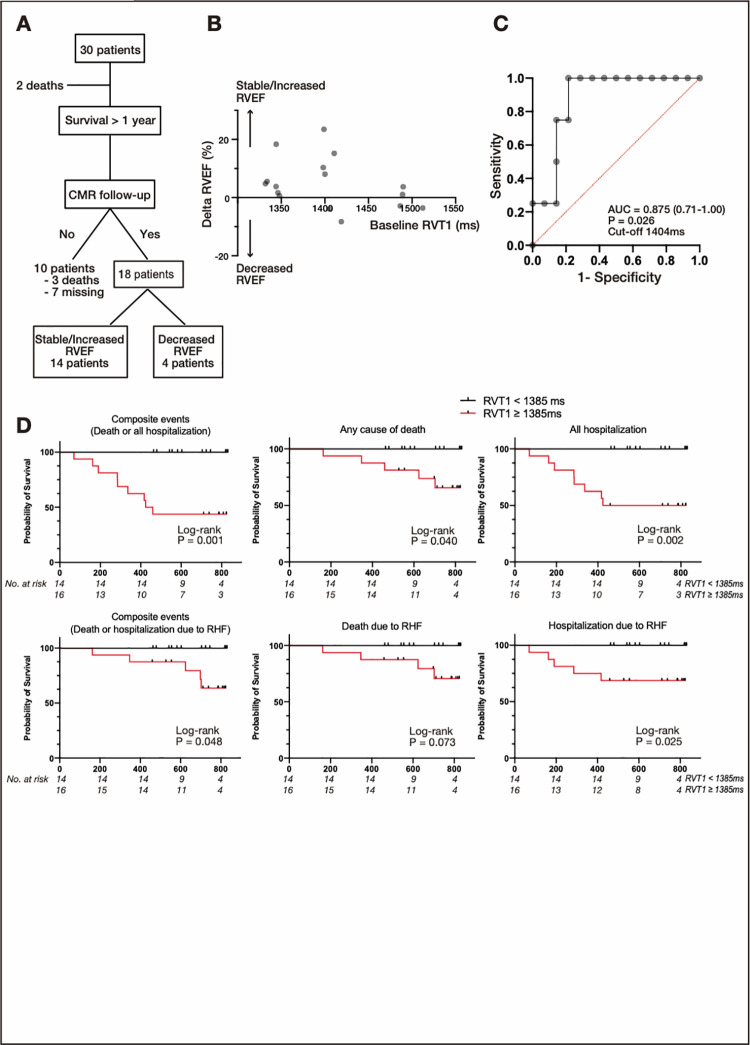
Predictive value of RVT1 for the decreased RV ejection fraction (RVEF) and poor outcome in pulmonary arterial hypertension (PAH). (a) Study profile. Thirty patients were enrolled (evaluating outcomes), and native T1 mapping using CMR and RHC was performed. Eighteen patients underwent volumetric CMR over a 12-month follow-up period (evaluating changes in RVEF). (b) Relationships between changes in the RVEF and baseline RVT1. (c) The receiver characteristics curve analysis for a decreased RVEF at follow-up. We defined a change of +3% or −3% (between baseline and follow-up over 12 months) as an increased or decreased RVEF, respectively. (d) Survival curves in 30 patients with PAH. Event-free survival was calculated according to native RVT1. Survival rates from events including composite outcomes of death and all hospitalizations, and death and hospitalization due to right heart failure, and each event were significantly reduced in patients with higher RVT1. Abbreviations: AUC, area under the curve; CMR, cardiovascular magnetic resonance; RHC, right heart catheterization; RHF, right heart failure; RVEF, right ventricular ejection fraction; RVT1, native T1 values of the right ventricular free wall.

A total of 18 patients underwent 12-month follow-up CMR examinations (Fig 2A). The RVEF did not improve in patients with higher RVT1 during the follow-up (Fig 2B). Patients with a decreased RVEF (n = 4, 22%) had higher RVT1 at baseline than did those with a stable/increased RVEF (n = 14, 78%; 1456±52 vs. 1381±57 ms, p = 0.033; Fig 2B, S2 Fig). The ROC analysis revealed that baseline RVT1 showed a remarkable predictive accuracy for a decreased RVEF (area under the curve: 0.87, 95% confidence interval [CI]: 0.71–1.00, p = 0.026, cut-off value: 1404 ms; Fig 2C).

### Survival of patients with pulmonary arterial hypertension

The median follow-up time was 703 (IQR: 551–810) days, and nine composite events (death or hospitalization from any cause) were observed. Four patients died due to right heart failure in the hospital and one at home.

The Cox proportional hazards model was applied to evaluate the significance of RVT1 using the T1 values of the septum, VIPs, and RV free wall. Multivariate analysis revealed that RVT1 was independently associated with composite events (hazard ratio: 1.02, 95% CI: 1.006–1.031, p = 0.002; S3 Table).

Moreover, the univariate Cox proportional hazards model was used to predict the time to composite events using the following 10 variables: age, sex, WHO-FC, disease duration, mean right atrial pressure, mean PAP, PVR, cardiac index, BNP levels, and RVT1 (Table 2).

**Table 2:** Cox proportional hazards analysis for composite events, including death or hospitalization from any cause.

Variables	Univariate analysis		Multivariate analysis	
	HR (95% CI)	p-values	HR (95% CI)	p-values
Age	1.011 (0.966–1.059)	0.635		
Female sex	0.467 (0.125–1.742)	0.257		
WHO-FC	2.897 (1.052–7.975)	0.04		
Disease duration	0.999 (0.996–1.003)	0.743		
Mean RAP	1.140 (1.035–1.257)	0.008		
Mean PAP	1.108 (1.027–1.195)	0.008		
PVR	1.045 (0.936–1.166)	0.433		
BNP	1.009 (1.003–1.015)	0.003		
Cardiac index	1.009 (0.500–2.034)	0.981		
T1 values of the RV free wall	1.019 (1.006–1.031)	0.002	1.019 (1.006–1.031)	0.002

Abbreviations: BNP, brain natriuretic peptide; CI, confidence interval; HR, hazard ratio; PAP, pulmonary arterial pressure; PVR, pulmonary vascular resistance; RAP, right atrial pressure; RV, right ventricle; WHO-FC, World Health Organization functional class.

Multivariate analysis with the forward selection method demonstrated that RVT1 was the only variable that was selected as a parameter associated with composite events. According to the mean RVT1 values of 1385 ms, Kaplan–Meier analyses showed that patients with higher RVT1 (≥ 1385 ms) had significantly poorer prognoses than did those with lower RVT1 (< 1385 ms) in terms of composite events, survival rates, and the incidence of hospitalizations including right heart failure related events (Fig 2D).

## Discussion

We employed a modified T1 mapping technique to evaluate myocardial T1 values of the RV free wall using 3.0-T CMR with a MOLLI sequence. We found that RVT1 was higher in patients with PAH compared to healthy controls, and compared to septal or VIP T1, RVT1 correlated strongly with RV performance, but not with hemodynamic parameters, in patients with PAH. Moreover, RVT1 was an independent predictor of composite outcomes including right heart failure related events. These findings suggest that native T1 mapping could be used to characterize advanced RV myocardial changes and has a prognostic potential in patients with PAH.

Right heart failure is the predominant cause of death in patients with PAH, and RV function is the most important determinant of prognosis [2]. However, conventional examinations of right heart remodeling have some limitations. For example, an endomyocardial biopsy is invasive, and the biopsy is generally procured from the RV septum, not from the RV free wall. LGE CMR is unable to detect diffuse pathological changes, and detecting the LGE contrast area with CMR imaging in the RV free wall is challenging considering its thin, crescent-like shape and the lack of normal surrounding myocardial tissues [22]. Notably, native T1 mapping, an emerging non-invasive CMR technique, assesses tissue characteristics without requiring contrast administration. However, the original evaluation method is challenging because of the partial volume effects of the thin walls and complex RV trabeculations. Thus, we modified the approach and acquired images in the end-systolic period using a motion-correction algorithm. This modified approach allows for analysis of the thickened and stabilized RV free wall, which enables the derivation of RVT1, during the systolic period. Moreover, we evaluated the inferior wall of the RV using ROIs because the inferior wall was thicker and more fixed than were other regions. Therefore, the modified approach greatly enhances the measurement reliability of RVT1.

Native T1 mapping quantifies the native T1 relaxation time per pixel; thus, tissue characteristics, such as lipid accumulation, myocardial edema, and fibrosis [13], can be assessed. Notably, a few studies have assessed T1 values in PAH. Native T1 values correlated with collagen area fraction based on the histological analysis of biopsy samples in patients with dilated cardiomyopathy [23] and were elevated at VIPs with increased interstitial collagen tissue and fiber disarray in an experimental PH porcine model with pulmonary vein banding [24]. Conversely, Roller et al. have reported significantly decreased septal T1 values with hemodynamic improvement 6 months after balloon pulmonary angioplasty in patients with chronic thromboembolic pulmonary hypertension, indicating that myocardial remodeling is partially reversible [15]. In the present study, T1 values of the septum, VIPs, and RV free wall were higher in patients with PAH compared with healthy controls, and the finding is consistent with that in a previous study [12]. We further found that T1 values of the RV free wall correlated positively with prolonged QRS duration, which is a predictor of RV fibrosis [25]. Taken together, these findings suggest that native T1 mapping could be used to characterize advanced RV myocardial changes. Further investigations are needed to clarify the association between RVT1 and histological changes using endomyocardial biopsies, explanted hearts, or autopsy materials.

To date, no study has evaluated the association between RVT1 and hemodynamic parameters [12, 26]. This study found that RVT1 did not correlate with hemodynamic parameters (mean PAP, cardiac index, and PVR) but were associated with BNP levels and CMR volumetric parameters (RVEF, RVESVi, and RVEDVi). Additionally, we analyzed both arterial load and ventricular performance independently using the single-beat method for a more in-depth understanding of RV performance. Interestingly, RVT1 correlated positively with Ea-pa/Ees-rv, a marker of RV-PA uncoupling, but negatively with Ees-rv, a marker of RV contractility. Patients with higher initial RVT1 showed a decreased RVEF over the 12-month follow-up period, which was related to poor prognosis. Hence, patients with higher RVT1 exhibited a failing, maladaptive state of RV function [27]. Moreover, we found that patients with higher RVT1 had worse clinical outcomes, and RVT1 was associated with composite events, independent of risk factors reported in current guidelines [3]. However, Saunders et al. have found that elevated native T1 values of the septum and VIPs did not contribute to the prediction of overall mortality [16]. This discrepancy could be due to the different T1 measurement sites. Moreover, LGE was observed in the septum or VIPs in 65–100% of patients with pulmonary hypertension and was not limited to severe cases [28, 29]. One possible reason is that the septum and VIPs might be affected by mechanical stress owing to septal wall bouncing, which is a sign of RV afterload and can be observed at an early stage [30]. Furthermore, studies found that the RVEDVi and RVEF were weakly correlated with VIP T1, but not with septal T1 [16], and the RV inferior free wall might be suitable to characterize the myocardium due to fewer motion artifacts [12]. Consistently, we identified stronger linear relationships of RVEDVi and RVEF with RVT1 than with septal or VIP T1, and RVT1 was more predictive than were septal and VIP T1 values. Therefore, the RV free wall would be more suitable than the septum or VIPs to measure native T1 values in patients with PAH. Further studies using RVT1 are needed to assess RV remodeling, and such studies would be essential for the future development of T1 mapping in PAH.

This study has several limitations. First, this was a single-center retrospective study with a relatively small number of patients. A larger cohort including each type of PAH is essential to validate the present findings since the pathophysiology of PAH varies between subtypes. Second, we did not investigate the correlation between T1 values and the degree of histological remodeling. Thus, future histological studies involving endomyocardial biopsies, explanted hearts, or autopsy materials are required. Third, RVT1 was assessed only at the inferior wall, not the entire free wall. In our preliminary study, T1 values of the overall free wall varied largely because of the partial volume effects of the surrounding tissues or the blood pool. Fourth, CMR measurements were performed using a 3.0-T system. Further research is needed to confirm our results with 1.5-T systems which is in more widespread use at present.

## Conclusions

Using native T1 mapping, we found that higher RVT1 was associated with BNP levels, RV performance, composite outcomes, and survival in patients with PAH. These findings suggest that native T1 mapping could be used to characterize advanced RV myocardial changes and has a prognostic potential in patients with PAH. Further global, multicenter studies are needed to validate the clinical utility of RVT1 mapping in patients with PAH.

## Supporting information

S1 Appendix Methods. (DOCX)

S1 Table Baseline T1 values of the septum, ventricular insertion points, and right ventricular free wall. Abbreviations: PAH, pulmonary arterial hypertension; RV, right ventricle.
(DOCX)

S2 Table Correlation coefficient between T1 values at each measurement site and clinical parameters. Abbreviations: BNP, brain natriuretic peptide; Ea-pa_i, effective pulmonary arterial elastance index; Ees-rv_i, effective right ventricular elastance index; PAP, pulmonary arterial pressure; PVR, pulmonary vascular resistance; RAP, right atrial pressure; RVEF, right ventricular ejection fraction; RVEDVi, right ventricular end-diastolic volume index; RVESVi, right ventricular end-systolic volume index; RV T1, native T1 values in the RV free wall; VIP, ventricular insertion point. *p<0.05, ^†^p<0.01, ^‡^p<0.001.
(DOCX)

S3 Table Cox proportional hazards analysis for composite events including death or hospitalization from any cause using t1 values of each measurement site. Abbreviations: CI, confidence interval; HR, hazard ratio; RV, right ventricle; VIPs, ventricular insertion points.
(DOCX)

S1 Fig Native T1 measurement using ROIs. Native T1 values were measured using regions of interest. Abbreviations: ROIs, regions of interests.
(DOCX)

S2 Fig Baseline RV T1 values of patients grouped by follow-up RVEF. According to a previous report, a +3% change defines an increased RVEF, while a −3% change defines a decreased RVEF at follow-up (see Methods). The baseline right ventricular (RV) T1 values were higher in patients with a decreased RVEF (n = 4) than in those with a stable/increased RVEF at follow-up examinations (n = 14). Abbreviations: RVEF, right ventricular ejection fraction.
(DOCX)
