# Supplementary material for: Prognostic value of right ventricular native T1 mapping in pulmonary arterial hypertension
Source: PLoS One. 2021 Nov 29;16(11):e0260456. doi: 10.1371/journal.pone.0260456 (PMC8629295; doi:10.1371/journal.pone.0260456)
Supplement: S1 Appendix — (DOCX) [file pone.0260456.s001.docx]

**Supporting Information**

**S1 Appendix**

**Methods**

**Data collection**

We obtained data pertaining to demographics, baseline characteristics, and medical treatment from patients’ medical records. All patients underwent the following evaluations: B-type natriuretic peptide (BNP) levels, chest radiography, electrocardiography, 6-minute walk test, right heart catheterization (RHC), and cardiac magnetic resonance recording. Mean pulmonary arterial wedged pressure, pulmonary artery pressure, right atrial pressure, cardiac output, and pulmonary vascular resistance were measured by RHC. Cardiac output was determined using Fick’s method and corrected for body surface area. BNP levels were measured from EDTA plasma samples using the automated immunoassay (AIA-CL2400, Tosoh Corp., Tokyo, Japan). The principal investigator had complete access to all the data in the study and is responsible for its integrity and analysis.

**Volumetric analysis using cardiac magnetic resonance imaging**

Cardiac magnetic resonance imaging was performed using a standardized clinical protocol with a 3.0-T system (MAGNETOM Verio and Vida; Siemens Healthcare GmbH, Erlangen, Germany). Left ventricular and right ventricular (RV) functions were assessed using cine imaging with a steady-state free precession sequence (trueFISP; echo time, 1.3 ms; repetition time, 2.6 ms; flip angle, 60°; slice thickness, 8 mm; gap width, 2 mm; and in-plane resolution, 4.1 × 2.7 mm^2^) by holding multiple breaths in contiguous short-axis and trans-axial slices that encompassed both ventricles and three standard long-axis slices. Prospective electrocardiogram gating was performed using the R-wave as a trigger. For quantification of RV end-diastolic volume (EDV), RV end-systolic volume (ESV), RV stroke volume, and right ventricular ejection fraction, two experienced radiologists manually traced the RV endocardial contours in the end-systolic and end-diastolic frames of trans-axial slices using a dedicated software program (Argus system; Siemens, Erlangen, Germany). Left ventricular volumes were determined similarly, although short-axis slices were used. EDV and ESV were corrected by body surface area as the EDV index and ESV index, respectively.
