# Supplementary material for: Prognostic value of right ventricular native T1 mapping in pulmonary arterial hypertension
Source: PLoS One. 2021 Nov 29;16(11):e0260456. doi: 10.1371/journal.pone.0260456 (PMC8629295; doi:10.1371/journal.pone.0260456)
Supplement: S1 Table — Abbreviations: PAH, pulmonary arterial hypertension; RV, right ventricle. (DOCX) [file pone.0260456.s002.docx]

**S1 Table**

| **Baseline T1 values of the septum, ventricular insertion points, and right ventricular free wall.** | | | | |
| --- | --- | --- | --- | --- |
|  |  | Healthy controls (n=16) | Patients with PAH (n=30) | p-values |
|  |  |  |  |  |
| T1 values (ms) | |  |  |  |
|  | Global LV | 1237±45 | 1334±102 | 0.001 |
|  | Septum | 1219±34 | 1350±91 | <0.001 |
|  | Ventricular insertion points | 1229±53 | 1434±118 | <0.001 |
|  | RV inferior free wall | 1226±54 | 1385±75 | <0.001 |
|  | | | | |

Abbreviations: LV, left ventricle; PAH, pulmonary hypertension; RV, right ventricle.
