# Supplementary material for: Prognostic value of right ventricular native T1 mapping in pulmonary arterial hypertension
Source: PLoS One. 2021 Nov 29;16(11):e0260456. doi: 10.1371/journal.pone.0260456 (PMC8629295; doi:10.1371/journal.pone.0260456)
Supplement: S2 Table — Abbreviations: BNP, brain natriuretic peptide; Ea-pa_i, effective pulmonary arterial elastance index; Ees-rv_i, effective right ventricular elastance index; PAP, pulmonary arterial pressure; PVR, pulmonary vascular resistance; RAP, right atrial pressure; RVEF, right ventricular ejection fraction; RVEDVi, right ventricular end-diastolic volume index; RVESVi, right ventricular end-systolic volume index; RV T1, native T1 values in the RV free wall; VIP, ventricular insertion point. *p<0.05, †p<0.01, ‡p<0.001. (DOCX) [file pone.0260456.s003.docx]

**S2 Table.**

**Correlation coefficient between T1 values at each measurement site and clinical parameters.**

| Variables | Septal T1 (R) | VIPs T1 (R) | RV T1 (R) |
| --- | --- | --- | --- |
| Mean RAP (mmHg) | 0.133 | 0.124 | 0.341 |
| Mean PAP (mmHg) | 0.471† | 0.336 | 0.249 |
| PVR (mmHg) | 0.269 | 0.238 | 0.081 |
| Cardiac index (L/min/m^2^) | −0.099 | −0.169 | 0.155 |
| RVEDVi (mL/m^2^) | 0.419* | 0.499† | 0.655‡ |
| RVESVi (mL/m^2^) | 0.429* | 0.473† | 0.637‡ |
| RV mass index | 0.476† | 0.301 | 0.622‡ |
| RVEF (%) | −0.340 | −0.217 | −0.420* |
| Ea-pa_i | 0.185 | −0.100 | −0.269 |
| Ees-rv_i | 0.169 | −0.179 | −0.539† |
| Ea-pa/Ees-rv | 0.376* | 0.234 | 0.457* |
| BNP (pg/mL) | 0.496† | 0.436* | 0.654‡ |
| QRS duration (ms) | 0.293 | 0.273 | 0.720‡ |
|  | | | |

Abbreviations: BNP, brain natriuretic peptide; Ea-pa_i, effective pulmonary arterial elastance index; Ees-rv_i, effective right ventricular elastance index; PAP, pulmonary arterial pressure; PVR, pulmonary vascular resistance; RAP, right atrial pressure; RVEF, right ventricular ejection fraction; RVEDVi, right ventricular end-diastolic volume index; RVESVi, right ventricular end-systolic volume index; RV T1, native T1 values in the RV free wall; VIP, ventricular insertion point.

*p<0.05, †p<0.01, ‡p<0.001.
