# Supplementary material for: Prognostic value of right ventricular native T1 mapping in pulmonary arterial hypertension
Source: PLoS One. 2021 Nov 29;16(11):e0260456. doi: 10.1371/journal.pone.0260456 (PMC8629295; doi:10.1371/journal.pone.0260456)
Supplement: S3 Table — Abbreviations: CI, confidence interval; HR, hazard ratio; RV, right ventricle; VIPs, ventricular insertion points. (DOCX) [file pone.0260456.s004.docx]

**S3 Table.**

**Cox proportional hazards analysis for composite events including death or hospitalization from any cause using T1 values of each measurement site.**

|  | Variables | Univariate analysis | | | | |  | | Multivariate analysis | | |
| --- | --- | --- | --- | --- | --- | --- | --- | --- | --- | --- | --- |
|  |  | HR (95% CI) | | p-values | | |  | HR (95% CI) | | p-values |  |
|  | T1 values of the septum | | 1.012 (1.004–1.020) | | 0.002 |  | |  | |  |  |
|  | T1 values of the VIPs | | 1.005 (0.999–1.010) | | 0.088 |  | |  | |  |  |
|  | T1 values of the RV free wall | | 1.019 (1.006–1.031) | | 0.002 |  | | 1.019 (1.006–1.031) | | 0.002 |  |

Abbreviations: CI, confidence interval; HR, hazard ratio; RV, right ventricle; VIPs, ventricular insertion points
