# Supplementary material for: Prognostic value of right ventricular native T1 mapping in pulmonary arterial hypertension
Source: PLoS One. 2021 Nov 29;16(11):e0260456. doi: 10.1371/journal.pone.0260456 (PMC8629295; doi:10.1371/journal.pone.0260456)
Supplement: S1 Fig — Native T1 values were measured using regions of interest. Abbreviations: ROIs, regions of interests. (DOCX) [file pone.0260456.s005.docx]

**S1 Figure.**

**Native T1 measurement using ROIs**

Native T1 values were assessed using regions of interest at septum, ventricular insertion points, and RV inferior free wall of the mid-ventricular short-axis T1 maps.
Abbreviations: ROIs, regions of interests
