# Supplementary material for: Prognostic value of right ventricular native T1 mapping in pulmonary arterial hypertension
Source: PLoS One. 2021 Nov 29;16(11):e0260456. doi: 10.1371/journal.pone.0260456 (PMC8629295; doi:10.1371/journal.pone.0260456)
Supplement: S2 Fig — According to a previous report, a +3% change defines an increased RVEF, while a −3% change defines a decreased RVEF at follow-up (see Methods). The baseline right ventricular (RV) T1 values were higher in patients with a decreased RVEF (n = 4) than in those with a stable/increased RVEF at follow-up examinations (n = 14). Abbreviations: RVEF, right ventricular ejection fraction. (DOCX) [file pone.0260456.s006.docx]

**S2 Figure.**

**Baseline RV T1 values of patients grouped by follow-up RVEF.**

According to a previous report, a +3% change defines an increased RVEF, while a −3% change defines a decreased RVEF at follow-up (see Methods). The baseline right ventricular (RV) T1 values were higher in patients with a decreased RVEF (n=4) than in those with a stable/increased RVEF at follow-up examinations (n=14).

Abbreviations: RVEF, right ventricular ejection fraction
